# Liposomal Bupivacaine Versus Ropivacaine in Combined Thoracic Paravertebral Block and Serratus Anterior Plane Block for Thoracoscopic Surgery: A Randomized Controlled Trial

**DOI:** 10.1002/kjm2.70258

**Published:** 2026-07-07

**Authors:** Lu Jin, Lian Tang

**Affiliations:** ^1^ Department of Anesthesiology Hunan Provincial People's Hospital, the First Affiliated Hospital of Hunan Normal University Changsha China

**Keywords:** liposomal bupivacaine, opioid‐sparing, serratus anterior plane block, thoracic paravertebral block, video‐assisted thoracoscopic surgery

## Abstract

The study aimed to compare the use of liposomal bupivacaine (LB) and ropivacaine hydrochloride (RH) in combined thoracic paravertebral block (TPVB) and serratus anterior plane block (SAPB) during video‐assisted thoracoscopic surgery (VATS). The primary outcome was cumulative consumption of 72‐h morphine milligram equivalent (MME) postoperatively. The secondary outcomes were MME consumption at 0–24 h, 24–48 h, and 48–72 h postoperatively, scores of visual analogue scale (VAS) at 6, 12, 24, 48, and 72 h postoperatively, effective times of patient‐controlled intravenous analgesia (PCIA), and first time to request for analgesia. The LB‐TPVB‐SAPB group (*n* = 66) exhibited a decrease in total MME consumption within 72 h postoperatively compared to the RH‐TPVB‐SAPB group (*n* = 66) [independent *t*‐test: *p* < 0.0001, Cohen's d = −1.05; mean difference with 95% confidence interval: −14.02 (−18.68 to −9.35)]. Bonferroni multiple comparisons test after repeated‐measures ANOVA showed less MME consumption at 0–24 h (*p* < 0.001) and 24–48 h (*p* = 0.013) with lower VAS scores at 24 (*p* < 0.001) and 48 h (*p* = 0.002) postoperatively in the LB‐TPVB‐SAPB group than the RH‐TPVB‐SAPB group. Fewer effective times of PCIA and longer time to first request for analgesia were also observed in the LB‐TPVB‐SAPB group than the RH‐TPVB‐SAPB group. In conclusion, this study demonstrates LB in combined TPVB and SAPB could reduce postoperative opioid consumption compared to RH in this combination after VATS and the absolute change in postoperative opioid consumption exceeds the reported minimal clinically important difference.

## 1 Introduction

Video‐assisted thoracoscopic surgery (VATS) techniques have revolutionized minimally invasive thoracic surgeries and become the mainstay for lung cancer surgery due to its important benefits in fewer injuries, less postoperative pain, and early mobilization compared to thoracotomy [1]. Even compared to robot‐assisted thoracic surgery (RATS), VATS yields comparable outcomes and allows more lymph node dissection [2]. Despite these advances, VATS still leads to moderate‐to‐severe acute postoperative pain with limited functional recovery in 12.7% of patients at 24 h postoperatively and 15.6% at 48 h postoperatively [3]. Opioids, such as morphine, fentanyl, and sufentanil, exert strong antinociceptive and analgesic effects and have been widely used in surgical settings for clinical anesthesia and postoperative analgesia. However, their use is associated with various adverse effects including postoperative nausea and vomiting, dizziness, respiratory depression, pruritus, chronic postsurgical pain, hyperalgesia, gastrointestinal paralysis, and opioid abuse [4]. For many anesthesiologists and thoracic surgeons, opioid‐sparing protocols to reduce postoperative opioid consumption while adequately addressing patients' pain remain a significant challenge.

Regional analgesia techniques have become important components of multi‐modal analgesia during VATS, among which thoracic paravertebral block (TPVB) and serratus anterior plane block (SAPB) are commonly employed techniques [5]. TPVB has become a standard regional anesthesia for VATS by accommodating local anesthetics to all surrounding spaces including intercostal, interpleural, epidural, and prevertebral spaces, providing unilateral somatic and sympathetic nerve blockade [6, 7]. SAPB is a novel regional anesthesia method to block the lateral cutaneous branch of the intercostal nerve, long thoracic nerve, and dorsal thoracic nerve by injecting local anesthetics to the serratus anterior space, which has increasingly been used in VATS as it can fully cover the surgical area affected by VATS [8]. In recent years, the combined use of TPVB and SAPB has gained popularity in VATS. Researchers believe this combination is safe, effective, and reliable for VATS due to its benefits in improving postoperative pain and reducing opioid consumption compared to TPVB or SAPB alone [9, 10]. At present, prolonging the duration of local anesthetics continues to be a significant challenge in TPVB [11]. Liposomal bupivacaine (LB) is a multivesicular formulation allowing extended local anesthetic release up to 72 h [12]. Recently, moderate evidence has demonstrated that the use of LB in peripheral nerve block could relieve postoperative pain for 3 days after all types of surgery compared to those using long‐acting local anesthetics including bupivacaine and ropivacaine [13]. In VATS, LB as a local anesthetic in TPVB or SAPB alone has presented clinical advantages over ropivacaine [14, 15]. However, there is limited evidence focusing on LB as a local anesthetic in the combination of TPVB and SAPB for VATS. The study aimed to investigate the effect of LB compared to ropivacaine hydrochloride (RH) in TPVB combined with SAPB on opioid consumption and postoperative pain after VATS. The researchers hypothesized that combined TPVB and SAPB with LB could effectively reduce postoperative opioid consumption and provide prolonged analgesia without adding adverse effects in patients after VATS.

## 2 Methods

### 2.1 Study Design

This is a prospective, randomized controlled trial study recruiting patients who were scheduled to undergo VATS for lung cancer at our hospital. Participant recruitment started in May 2024 and ended in July 2025. The study was performed in strict accordance with the principles of the Declaration of Helsinki and approved by the Ethics Committee of our hospital. All participants provided written informed consent.

### 2.2 Inclusion and Exclusion Criteria

The inclusion criteria were patients pathologically diagnosed as primary lung cancer, patients undergoing elective VATS under general anesthesia, patients with an American Society of Anesthesiologists (ASA) physical status of 1 to 3, and patients aged ≥ 18 years. Patients were excluded if they were allergic to local anesthetics used in the study, had severe coagulation dysfunction, cirrhosis, renal impairment, severe hypertension, known mental illness, or malignancy other than primary lung cancer, a significant psychiatric history (major depression or generalized anxiety disorder), preexisting chronic pain (lasting for ≥ 3 months), or daily use of opioid analgesics or history of opioid abuse. Those with conversion to open thoracotomy or experiencing severe surgical complications during the perioperative period were also excluded.

### 2.3 Sample Size Calculation

In a preliminary pilot study performed at our hospital between March and April in 2024, cumulative consumption of 72‐h morphine milligram equivalent (MME) after VATS was 90.44 ± 14.62 mg in the RH‐TPVB‐SAPB group and 80.72 ± 13.19 mg in the LB‐TPVB‐SAPB group (*n* = 10 per group). The sample size was calculated by using the G*power software (version 3.1.9.2) based on total MME consumption. With α = 0.05 and power = 0.95 in a two‐tailed test, a minimum of 55 participants per group was needed. Considering an anticipated 20% dropout, the target sample size was increased to 66 per group, resulting in a sample size of 132 participants in total.

### 2.4 Randomization and Blinding

Participants were randomly assigned to the LB‐TPVB‐SAPB group or the RH‐TPVB‐SAPB group in a 1:1 ratio by simple randomization. An independent researcher outside the trial generated 132 random numbers using Microsoft Excel to perform randomization. Each random number corresponding to one participant was placed into sealed, opaque envelopes. A nurse outside the trial opened envelopes in the order of participant enrollment and prepared study medications in identical syringes containing local anesthetic and labeled “solution A” or “solution B” to minimize visual clues from solution appearance. In this trial, the participants, intervention providers, outcome assessors, and data analysts were all unaware of group assignments until all statistical analyses were completed.

### 2.5 Ultrasound‐Guided Block Interventions

Upon entering the operating room, each patient was positioned laterally to receive TPVB and SAPB before general anesthesia. The TPVB was performed at the paravertebral space 2 cm from the midline of the spine and the upper and lower adjacent paravertebral space where the planned surgical incision was located and guided by an ultrasound machine (SonoScape, Shenzhen, China). The transducer (5–10 MHz low‐frequency) was perpendicular to the intraplane approach of the spine to visualize the paravertebral space. After skin disinfection, a 21‐G nerve puncture needle was inserted deep into the triangular space formed by the pleural parietal layer, intercostal lining, and tip of the transverse process under ultrasound guidance. Upon confirming the absence of blood or cerebrospinal fluid via aspiration, a single injection of 20 mL of local anesthetic solution (either LB or RH) was administered. Then, the transducer was placed against the midaxillary line to clearly visualize the pleura, the lateral arch of the 4th or 5th rib and the different muscle planes from the most superficial to the deepest: the latissimus dorsi muscle, the serratus anterior muscle, and the intercostal muscles. The needle was inserted in the plane of the transducer into the 4th or 5th intercostal space, and the progress of the needle was followed on the screen until it reached the space between the serratus anterior muscle and the intercostal muscles. Upon confirming negative aspiration, a single injection of 20 mL of local anesthetic solution (either LB or RH) was administered. The prescription of local anesthetic solution was as follows: LB was prepared by diluting 10 mL of EXPAREL (133 mg, Pacira Biosciences, San Diego, CA, USA) with 10 mL of normal saline to yield a total volume of 20 mL; RH was prepared by diluting 10 mL of 0.375% ropivacaine solution with 10 mL of normal saline to yield a total volume of 20 mL. All nerve block procedures were performed by the same experienced anesthesiologist. The effectiveness of the blocks was evaluated by an anesthesiologist blinded to the group allocation, with pinprick sensation every 5 min in each dermatomal distribution from T3 to T7. [Correction added on 14 July 2026, after first online publication: The manufacturer name for EXPAREL has been revised in this version.]

### 2.6 General Anesthesia

Induction of anesthesia was performed by using 0.02 mg/kg midazolam, target‐controlled infusion of propofol at a plasma target concentration of 4.0 μg/mL, 0.5 μg/kg sufentanil, and 0.2–0.3 mg/kg cisatracurium. Anesthesia was maintained by administering propofol at a rate of 4–10 mg/kg/h and remifentanil at a rate of 0.1–0.3 μg/kg/min, with bispectral index (BIS) maintained at 40–60.

### 2.7 Analgesia Protocol

After surgery, all patients received the patient‐controlled intravenous analgesia (PCIA) and the instructions for the PCIA pump were performed by the nurse blinded to the group assignments. The PCIA consisted of 100 μg sufentanil and 16 mg ondansetron hydrochloride diluted to 100 mL with 0.9% normal saline and was configured as no background infusion, a bolus amount of 2 mL, a locking time of 15 min, and a maximum hourly dose of 10 mL. The pain degree of patients was evaluated using the VAS, and 3 mg of morphine was intramuscularly administered as a rescue analgesic when the patients reported intolerable pain or their pain was scored ≥ 4 on the VAS.

### 2.8 Outcome Measures

The primary outcome was cumulative consumption of 72‐h MME. The postoperative opioid consumption included PCIA use and rescue intramuscular morphine within 72 h postoperatively, which was converted to total MMEs. The MME was considered the equivalent of 0.5 μg of intravenous sufentanil [16]. The secondary outcomes were MME consumption at 0–24 h, 24–48 h, and 48–72 h postoperatively, the scores of VAS at 6, 12, 24, 48, and 72 h postoperatively, the effective times of PCIA, the time of first request for analgesia, the incidence of adverse effects (hypotension, bradycardia, nausea and vomiting, and dizziness), length of hospital stay, and length of postoperative hospital stay.

### 2.9 Statistical Analysis

The normality of the distributions of continuous variables was evaluated using the Shapiro–Wilk test. Descriptive statistics were performed to present a summary of continuous variables. When normal distribution was confirmed, continuous variables were summarized in the form of mean ± standard deviation (s.d.) and group differences were analyzed by the independent *t*‐test. When being out of normal distribution, continuous variables were summarized in the form of median with interquartile range (IQR; Q1: 25th percentile, Q3: 75th percentile) and group differences were analyzed by the Mann–Whitney U test. Categorical variables are reported as numbers with percentages (%) and analyzed by using the chi‐squared tests. The MME consumption at 0–24 h, 24–48 h, and 48–72 h postoperatively, the scores of VAS at 6, 12, 24, 48, and 72 h postoperatively were analyzed by using repeated‐measures analysis of variance (ANOVA) followed by multiple comparisons test (Bonferroni correction). All statistical tests used a two‐tailed *p* < 0.05 as statistically significant in GraphPad prism, version 8.0 (GraphPad, San Diego, CA, USA).

## 3 Results

**FIGURE 1:**
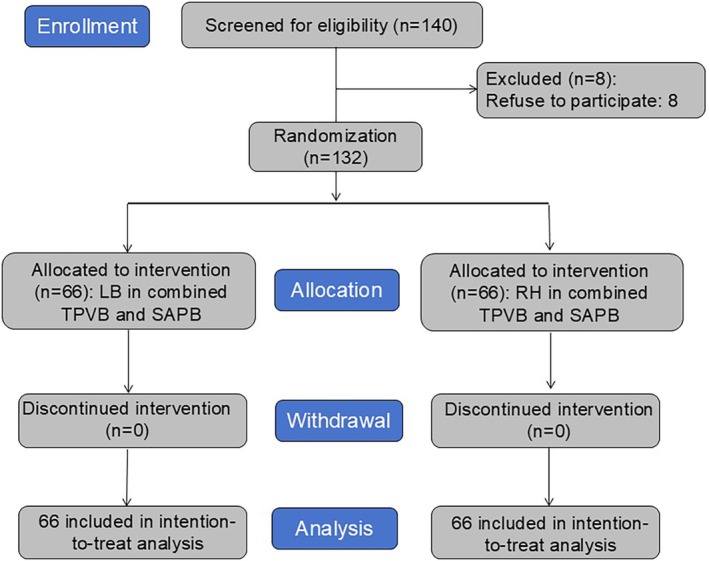
The flowchart of participant recruitment. LB, liposomal bupivacaine; RH, ropivacaine hydrochloride; SAPB, serratus anterior plane block; TPVB, thoracic paravertebral block.

A total of 140 patients undergoing VATS were initially screened for eligibility, and 8 were excluded due to their refusal to participate in this trial. A total of 132 patients were included in the intention‐to‐treat analysis (66 per group) (Figure 1). All patients were successfully blocked. No failed blocks, incomplete blocks, or repeated blocks were found in this study. Demographic, surgical, and blocking characteristics of participants were well‐balanced and comparable between the LB‐TPVB‐SAPB group and the RH‐TPVB‐SAPB group (Table 1).

**TABLE 1:** Demographic and surgical characteristics of participants.

Characteristic	LB‐TPVB‐SAPB (*n* = 66)	RH‐TPVB‐SAPB (*n* = 66)	*p*
Age (year, mean ± s.d.)	55.45 ± 7.91	56.76 ± 8.04	0.350
Sex (male, *n*/%)	34 (51.52%)	39 (59.09%)	0.484
BMI (kg/m^2^, mean ± s.d.)	24.22 ± 3.28	24.82 ± 3.22	0.285
ASA classification [I/II, *n* (%)]	26/40 (39.39%/60.61%)	30/36 (45.45%/54.55%)	0.598
Comorbid hypertension [yes, *n* (%)]	20 (30.30%)	25 (37.88%)	0.463
Comorbid diabetes mellitus [yes, *n* (%)]	5 (7.58%)	3 (4.55%)	0.718
Surgical procedure [*n* (%)]			0.520
Lobectomy	36 (54.55%)	30 (45.46%)	
Segmentectomy	20 (30.30%)	22 (33.33%)	
Wedge resection	10 (15.15%)	14 (21.21%)	
Surgery time [min, mean ± s.d.]	131.60 ± 24.96	135.10 ± 30.51	0.472
Number of blocked dermatomes [median (Q1, Q3)]	5 (4, 7)	5 (4, 6)	0.360
Intraoperative sufentanil consumption [μg, median (Q1, Q3)]	30 (25, 30)	30 (25, 30)	0.721
Intraoperative remifentanil [mg, median (Q1, Q3)]	0.67 (0.52, 0.86)	0.71 (0.50, 0.89)	0.389
Use of acetaminophen [*n* (%)]	35 (53.03%)	33 (50.00%)	0.728

*Note:* When data were described as mean ± s.d., the independent *t*‐test was used. When data were described as numbers (percentage), the chi‐square test was used. When data were described as median (Q1, Q3), the Mann–Whitney U test was used.

Abbreviations: ASA, American Society of Anesthesiologists; BMI, body mass index; LB, liposomal bupivacaine; RH, ropivacaine hydrochloride; SAPB, serratus anterior plane block; TPVB, thoracic paravertebral block.

**FIGURE 2:**
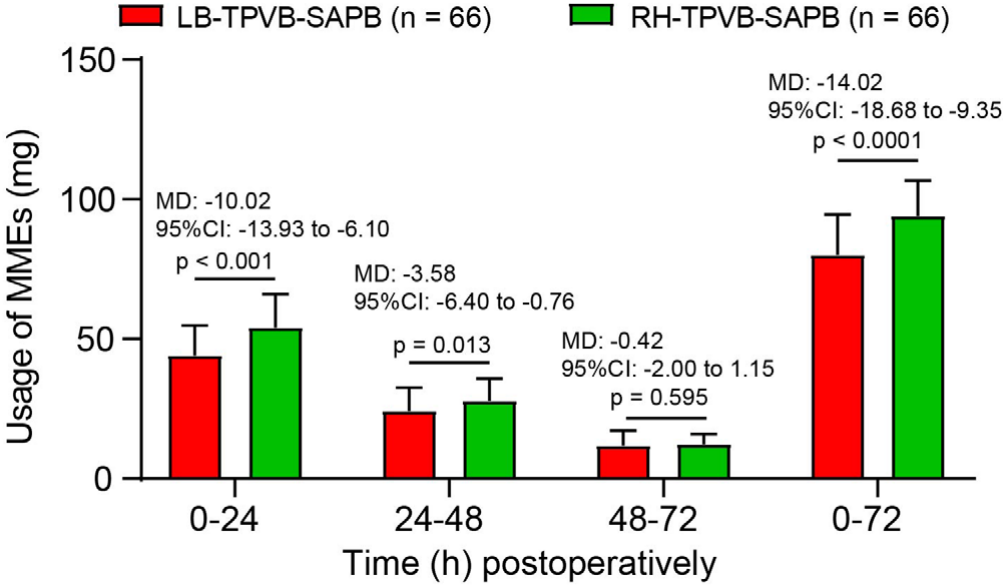
The MME consumption at 0–24 h, 24–48 h, and 48–72 h postoperatively, and the cumulative consumption of 72‐h MME postoperatively between the LB‐TPVB‐SAPB group and the RH‐TPVB‐SAPB group. Data summarized as mean ± s.d. 95% CI, 95% confidence interval; LB, liposomal bupivacaine; MD, mean difference; MME, morphine milligram equivalent; RH, ropivacaine hydrochloride; SAPB, serratus anterior plane block; TPVB, thoracic paravertebral block.

The LB‐TPVB‐SAPB group had a decrease in cumulative consumption of 72‐h MME postoperatively compared to the RH‐TPVB‐SAPB group (independent *t*‐test: *p* < 0.0001, Cohen's d = −1.05, Figure 2). The repeated‐measures ANOVA indicated significant treatment × time interactions in MME consumption at 0–24 h, 24–48 h, and 48–72 h postoperatively (F = 10.12, *p* < 0.001, partial η^2^ = 0.072; Figure 2). Further Bonferroni multiple comparisons test showed the LB‐TPVB‐SAPB group had decreases in MME consumption at 0–24 h (*p* < 0.001) and 24–48 h (*p* = 0.013). No significant difference in the MME consumption at 48–72 h was found between LB‐TPVB‐SAPB and RH‐TPVB‐SAPB groups (*p* = 0.595).

**FIGURE 3:**
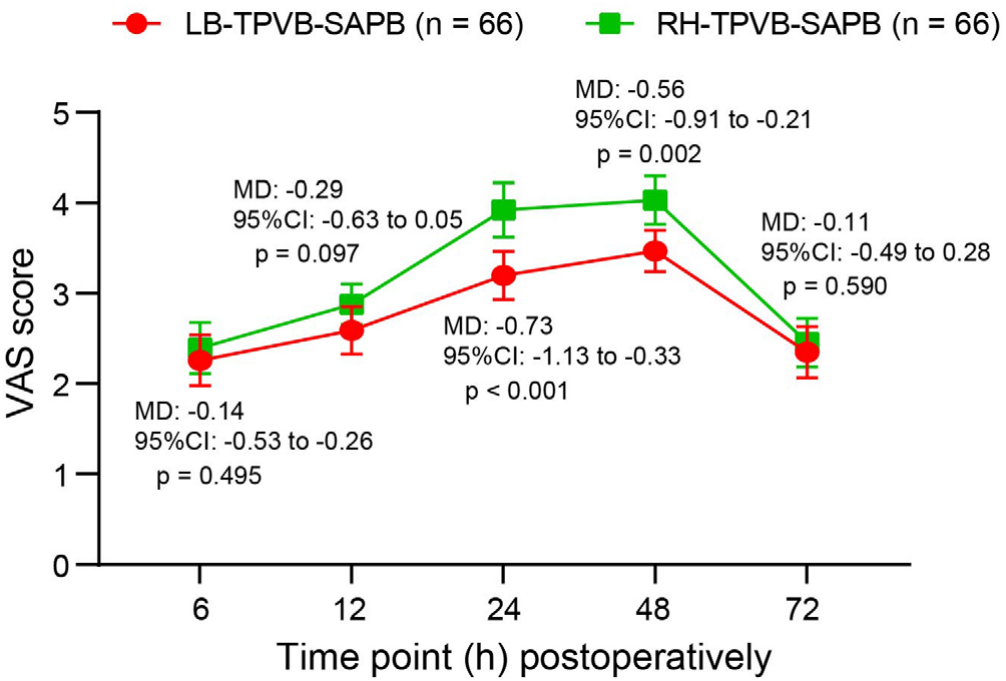
The VAS scores at predetermined intervals (6, 12, 24, 48, and 72 h postoperatively) between the LB‐TPVB‐SAPB group and the RH‐TPVB‐SAPB group. Data summarized as mean with 95% CI. 95% CI, 95% confidence interval; LB, liposomal bupivacaine; RH, ropivacaine hydrochloride; SAPB, serratus anterior plane block. MD, mean difference; TPVB, thoracic paravertebral block; VAS, visual analogue scale.

The repeated‐measures ANOVA indicated significant treatment × time interactions in VAS scores at 6, 12, 24, 48, and 72 h postoperatively (F = 2.72, *p* = 0.038, partial η^2^ = 0.021; Table 2 and Figure 3). Further Bonferroni multiple comparisons test showed lower scores of VAS at 24 (*p* < 0.001) and 48 h (*p* = 0.002) postoperatively in the LB‐TPVB‐SAPB group than the RH‐TPVB‐SAPB group (Table 2 and Figure 3). No significant difference in VAS scores at other time points postoperatively was observed between LB‐TPVB‐SAPB and RH‐TPVB‐SAPB groups (*p* > 0.05).

**TABLE 2:** The median with ranges of VAS scores at 6, 12, 24, 48, and 72 h postoperatively between the LB‐TPVB‐SAPB group and the RH‐TPVB‐SAPB group.

	LB‐TPVB‐SAPB (*n* = 66)	RH‐TPVB‐SAPB (*n* = 66)	*p*
VAS score, mean (95% CI)			
Postoperative 6 h	2.26 (1.98–2.54)	2.39 (2.11–2.68)	0.495
Postoperative 12 h	2.59 (2.33–2.85)	2.88 (2.66–3.10)	0.097
Postoperative 24 h	3.20 (2.93–3.46)	3.92 (3.63–4.22)	< 0.001
Postoperative 48 h	3.47 (3.24–3.70)	4.03 (3.77–4.30)	0.002
Postoperative 72 h	2.35 (2.06–2.63)	2.46 (2.19–2.72)	0.590

*Note:* Repeated‐measures ANOVA followed by multiple comparisons test (Bonferroni correction) was used for statistical analysis.

Abbreviations: 95% CI, 95% confidence interval; LB, liposomal bupivacaine; RH, ropivacaine hydrochloride; SAPB, serratus anterior plane block; TPVB, thoracic paravertebral block; VAS, visual analogue scale.

Fewer effective times of PCIA with a longer time to first request for analgesia were also found in the LB‐TPVB‐SAPB group than the RH‐TPVB‐SAPB group (Table 3). Two groups did not differ in the incidence of adverse effects, length of hospital stay, and length of postoperative hospital stay (*p* > 0.05, Table 3).

**TABLE 3:** Other secondary outcomes between the LB‐TPVB‐SAPB group and the RH‐TPVB‐SAPB group.

	LB‐TPVB‐SAPB (*n* = 66)	RH‐TPVB‐SAPB (*n* = 66)	*p*
Effective time of PCIA (mean ± s.d.)	16.52 ± 2.82	17.83 ± 3.01	0.010
Time to first request for analgesia [h, median (Q1, Q3)]	23.50 (15.25, 35.75)	14.00 (8.00, 25.00)	0.003
Adverse effects [*n* (%)]	18 (27.27%)	24 (36.36%)	0.350
Nausea and vomiting	4	4	
Dizziness	3	5	
Hypotension	6	8	
Bradycardia	5	7	
Length of hospital stay [d, median (Q1, Q3)]	10.50 (8.00, 13.00)	11.50 (8.00, 14.00)	0.198
Length of postoperative hospital stay [d, median (Q1, Q3)]	8.00 (7.00, 10.00)	9.00 (6.00, 11.00)	0.531

*Note:* When data were described as mean ± s.d., the independent *t*‐test was used. When data were described as numbers (percentage), the chi‐square test was used. When data were described as median (Q1, Q3), the Mann–Whitney U test was used.

Abbreviations: LB, liposomal bupivacaine; PCIA, patient‐controlled intravenous analgesia; RH, ropivacaine hydrochloride; SAPB, serratus anterior plane block; TPVB, thoracic paravertebral block.

## 4 Discussion

This prospective, randomized controlled trial study demonstrated that the use of LB in TPVB combined with SAPB could reduce 72‐h MME postoperatively without adding adverse effects for patients undergoing VATS compared to the use of RH in this combination.

The opioid‐sparing anesthetic protocol for perioperative analgesia has been largely successful with the use of local anesthetics during procedures such as peripheral nerve blocks [17]. SAPB and TPVB have different mechanisms of action, and two of them complement each other well. SAPB targets the lateral cutaneous branches of the intercostal nerves which cover the anterior chest wall, lateral chest wall, and posterior chest wall, providing extensive analgesic effects on one side of the chest wall nerves [18]. However, SAPB provides limited sympathetic blockade. TPVB targets the dorsal, ventral, sympathetic, and communicating branches of the intercostal nerves to produce spinal and sympathetic nerve blockade, providing extensive analgesic effects to deeper chest structures and offering a more comprehensive blockade [19]. This combination effectively compensates for the individual limitations of each block for VATS, offering more improved postoperative pain, reducing opioid usage, and shortening the length of hospital stay [10].

Previous evidence has shown promising benefits of LB in postoperative pain control and opioid savings without adding significant adverse effects in multiple surgical settings, including thoracoscopic lung surgery [20]. LB is derived from bupivacaine hydrochloride encapsulated within multiple, non‐concentric lipid bi‐layers, which allows rapid absorption of bupivacaine and prolonged delivery to the target site [21]. Prolonged release of LB provides adequate and sustained peripheral nerve block, which can prevent or reduce the transmission of nociceptive signals caused by surgical trauma to the central nervous system, thereby reducing central sensitization to prevent post‐surgical pain [22]. The analgesic effect of RH may have begun to decline early post‐surgery, while LB provides a sustained block that may cover the peak period of pain. These may explain the statistically significant observations of reduced 72‐h MME consumption postoperatively and decreased VAS scores at 24 and 48 h postoperatively in the LB‐TPVB‐SAPB group compared to the RH‐TPVB‐SAPB. Similar results have been obtained with the use of LB comparing with ropivacaine for peripheral nerve blocks in managing postoperative pain following thoracoscopic surgery [23, 24]. In this study, the absolute change in 72‐h MME consumption between the LB‐TPVB‐SAPB group and the RH‐TPVB‐SAPB group was 14.02 mg, and this change exceeded the reported minimal clinically important difference (MCID) (MME consumption: 10 mg) [25]. However, the absolute changes in VAS scores at 24 and 48 h postoperatively between two groups were −0.73 and −0.56, which achieved statistically significant difference but did not exceed the reported MCID (VAS scores: 1.5 at rest) [26]. Overall, the prolonged analgesia provided by LB‐TPVB‐SAPB compared to RH‐TPVB‐SAPB should be further proven by use of VAS scale (0–100 mm).

Several limitations in the present study should be noted. First, the single‐center design with a limited small sample size may constrain the generalizability of our results to broader populations. Possible multi‐center trials with larger‐scale populations are needed to optimize the application strategies of this dual‐block technique. Second, the sample size calculation was based on a pilot study with only 10 patients per group, which may overestimate the effect size. Third, the observed benefit of liposomal bupivacaine may be attributable not only to its prolonged‐release characteristics but also to differences in total local anesthetic exposure compared with the ropivacaine regimen used in this study; therefore, LB warrants further investigation for its potential to reduce chronic postsurgical pain risk after VATS compared to RH or its opioid‐sparing effect compared to a higher dose of ropivacaine. Fourth, the substantial cost disparity between LB and RH necessitates formal health‐economic evaluations and comparative cost‐utility analyses to determine economic feasibility in resource‐limited settings, despite potential hospitalization cost reductions from decreased opioid use. Fifth, the pain score difference being statistically significant but not exceeding the reported MCID may cause caution in the use of LB or applying this dual‐block technique.

In summary, the study demonstrates the combined implementation of TPVB and SAPB using LB as a local anesthetic could confer an opioid‐sparing benefit in the surgical setting of VATS. Considering the critical component of opioid management in the setting of enhanced recovery after surgery (ERAS) protocols, this study may coincide well with the unique value for the role of this dual‐block technique with LB within ERAS protocols during VATS.

## Conflicts of Interest

The authors declare no conflicts of interest.

## Data Availability

All data generated or analyzed during this study are included within the manuscript.
